# Interpreting Null Findings from Trials of Alcohol Brief Interventions

**DOI:** 10.3389/fpsyt.2014.00085

**Published:** 2014-07-16

**Authors:** Nick Heather

**Affiliations:** ^1^Department of Psychology, Faculty of Health and Life Sciences, Northumbria University, Newcastle upon Tyne, UK

**Keywords:** alcohol-related problems, brief interventions, randomized controlled trials, null findings, null hypothesis significance testing, Bayesian statistics

## Abstract

The effectiveness of alcohol brief intervention (ABI) has been established by a succession of meta-analyses but, because the effects of ABI are small, null findings from randomized controlled trials are often reported and can sometimes lead to skepticism regarding the benefits of ABI in routine practice. This article first explains why null findings are likely to occur under null hypothesis significance testing (NHST) due to the phenomenon known as “the dance of the *p*-values.” A number of misconceptions about null findings are then described, using as an example the way in which the results of the primary care arm of a recent cluster-randomized trial of ABI in England (the SIPS project) have been misunderstood. These misinterpretations include the fallacy of “proving the null hypothesis” that lack of a significant difference between the means of sample groups can be taken as evidence of no difference between their population means, and the possible effects of this and related misunderstandings of the SIPS findings are examined. The mistaken inference that reductions in alcohol consumption seen in control groups from baseline to follow-up are evidence of real effects of control group procedures is then discussed and other possible reasons for such reductions, including regression to the mean, research participation effects, historical trends, and assessment reactivity, are described. From the standpoint of scientific progress, the chief problem about null findings under the conventional NHST approach is that it is not possible to distinguish “evidence of absence” from “absence of evidence.” By contrast, under a Bayesian approach, such a distinction is possible and it is explained how this approach could classify ABIs in particular settings or among particular populations as either truly ineffective or as of unknown effectiveness, thus accelerating progress in the field of ABI research.

The effectiveness of alcohol brief intervention (ABI) in reducing alcohol consumption among hazardous and harmful drinkers is generally considered to have been demonstrated by a succession of systematic reviews with meta-analysis (1). The focus of these reviews in terms of types of ABI and settings for implementation has varied, together with the precise form in which effectiveness has been demonstrated (e.g., with regard to the intensity of effective intervention) (2, 3). The conclusions of secondary analyses concerning, for example, gender differences in response to ABI (2, 4) have also differed. There is little good evidence as yet for the effects of ABI on outcomes beyond consumption, e.g., morbidity or mortality (5). While apparently strong in the primary health care (PHC) setting, the evidence to support ABI in emergency (6) and general hospital (7) settings is more equivocal. But despite these reservations, all meta-analytic reviews of ABI in general and ABI in PHC in particular have found, without exception, that participants who receive ABI show greater reductions in alcohol consumption at follow-up than those who do not.

This positive verdict on the effectiveness of ABI notwithstanding, null findings from randomized or otherwise controlled trials, in which the statistical superiority of ABI over control conditions has not been demonstrated, frequently occur; they are often encountered in the literature and routinely reported at scientific conferences. Given the overall benefits of ABI shown in meta-analyses, reasons for these frequent failures to confirm effectiveness are not obvious but it may be that the effects of ABI are sufficiently small that they are difficult to detect (see below), in addition to other possible reasons. Whatever the reasons, they can have a dispiriting effect on researchers, health care administrators, and policy-makers. Researchers may be discouraged from pursuing research in the field of ABI and may not bother to submit their null findings for publication (8). Even if papers reporting null findings are submitted, and despite frequent admonitions that null findings based on competently designed research should be published (9), they may be rejected by journal editors, thus possibly biasing the results of meta-analyses. Health administrators may be persuaded to devote more resources to other areas of health care and policy-makers may listen more sympathetically to the arguments of those who are opposed to the widespread implementation of ABI as a means of reducing alcohol-related harm in the population (10). The damaging effects of null findings may be especially pronounced when they originate from large, expensively funded, and well-publicized trials.

Another kind of problem associated with null findings is that they may be misinterpreted, leading sometimes to inappropriate calls for the implementation of interventions that lack supporting evidence. A prominent source of such misinterpretation arises because of the classic error of “proving the null hypothesis.” Confusion is also likely to arise because of the frequent finding in trials of ABI of reductions in drinking, sometime quite large, in control conditions. Lastly, a limitation of the interpretation of null findings under the conventional null hypothesis significance testing (NHST) approach to ABI research is that it is unable to distinguish between two potentially different conclusions: that there is no evidence that the intervention under study is effective and that there is evidence that it is ineffective. As we shall see, this limitation has a retarding effect on scientific progress in this area of research.

Against this background, the issue of null findings from trials of ABI will be discussed with the following aims:

- To show that, even though effects of ABI in the population may be real, it is not surprising that these effects often fail to be detected in research trials.
- To describe ways in which null findings are often misunderstood, with potentially damaging consequences for practice and policy on ABI.
- To explore one of the key characteristics of null findings in the field of ABI research – the tendency for control groups to show relatively large reductions in alcohol consumption.
- To suggest a way in which one of main drawbacks arising from null findings – the inability to distinguish between “absence of evidence” and “evidence of absence” – can be overcome.

## The Dance of the *p*-Values

Over the past few years a YouTube video presentation by Emeritus Professor Geoff Cumming of La Trobe University, Melbourne, VIC, Australia, entitled “The dance of the *p*-values,”^1^ has been circulating universities around the world [see also Ref. (11), p. 135–42]. Cumming amusingly and persuasively illustrates the enormous variability in the *p*-value simply due to sampling variability. He claims that most researchers fail to appreciate how unreliable the *p*-value is as a measure of the strength of evidence to support a finding.

**Figure 1:**
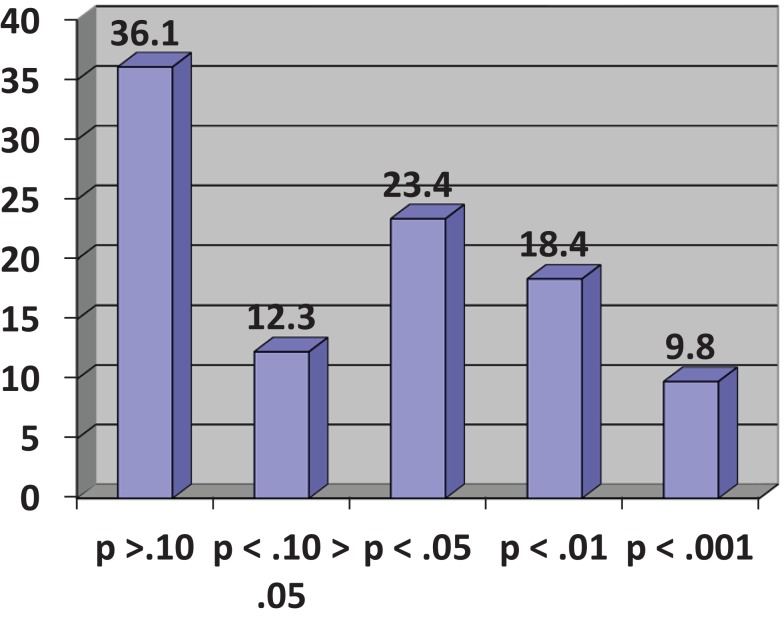
**Frequency histogram of *p*-values (%) for 1,500 simulated experiments (see text)**. Adapted from Cumming ((11), p. 139).

In his demonstration, Cumming considers an experiment consisting of two independent groups, Experimental (E) and Control (C), designed to investigate the effect of an intervention on a variable measuring some relevant participant behavior. He assumes a population effect of the intervention, unknown of course to the experimenter, equivalent to an effect size of half a standard deviation or Cohen’s δ = 0.5, conventionally regarded as a medium effect (12). This results in two normally distributed populations with standard deviations of the same size. In the experiment, both E and C groups have size *N* = 32, giving a power to detect a medium-sized effect of 0.52 for a two-tailed test with α = 0.05. Using his *Explanatory Software for Confidence Intervals* (ESCI)^2^, Cumming runs a simulation of 1,500 experiments by sampling from the assumed populations and observes the resulting distribution of *p*-values for the obtained differences between E and C group means. These range from *p* = 0.8 to *p* < 0.001, even though there has been no change in the population effect. When grouped in a frequency histogram (Figure 1), the most frequent category of *p*-values at 36.1% is those exceeding *p* = 0.10 and clearly non-significant. A further 12.3% are in the questionable, “approaching significance” range of between *p* <0.10 and >0.05. Altogether, 48.4% of *p*-values are >0.05, meaning that by orthodox statistical practice on nearly half the occasions this experiment might be conducted a null finding would eventuate, even though there is an effect of intervention in the population. The other 51.6% of results would be taken as statistically significant but these are distributed over the conventional labels of “significant” (*p* < 0.05), “highly significant” (*p* < 0.01), and “very highly significant” (*p* < 0.001), even though, again, nothing has changed in the size of the effect in the population. Cumming likens running a single experiment under these circumstances to visiting “the *p*-value casino” because the obtained *p*-value will be randomly chosen from the infinite series of possible values; obtaining a statistically significant *p*-value is like winning at roulette. The calculation of effect sizes with confidence intervals gives much more reliable information on what is likely to happen on replication (13).

It might be objected here that randomized controlled trials of ABI are usually more powerful than the experiment in the preceding paragraph. This may be true, although sample sizes not much different from *N* = 32 per group are not unknown in the scientific literature on ABI. Against that, the effect size for ABI is likely to be smaller than δ = 0.5 and is better estimated as small to medium (14), say δ = 0.35. The distribution of possible *p*-values from any given experiment depends solely on statistical power. If the conventional recommendation for adequate power of 80% is accepted, in a two-group comparison similar to that described above, the sample size necessary to detect a small to medium effect by a two-tailed *t*-test at the 5% significance level and assuming equally sized groups is 130 per group [G*Power 3.0.10, (15)]. A minority of trials of ABI are this big and the remainder will be subject to varying degrees to the casino scenario described above. Even with a power of 80% to detect a real but small to medium effect, one-fifth of possible *p*-values will fail to reach the 0.05 significance level and will be erroneously regarded as null findings, i.e., they will be Type II errors. If the assumption of the effect of ABI is made more conservatively at δ = 0.2, conventionally regarded as a small effect and arguably a minimally interesting effect of ABI, a sample size of 394 per group is needed to give a 80% chance of detecting an effect and very few trials of ABI are this large.

The solution to this problem of widely varying *p*-values carrying little information is, according to Cumming (11) and to many others, to abandon NHST in favor of estimating effect sizes with confidence intervals. He points out that this estimation approach to research findings is standard in the “hard” sciences like physics and chemistry, is commonly employed in most areas of medical research, and has been recommended in the Publications Manual of the *American Psychological Association* (16). At the same time, NHST has been severely criticized now for over 50 years (17) but still continues to be popular and standard practice in many disciplines within the human sciences. Without attempting to resolve this issue here, what can be said is that the abandonment of NHST – and particularly the abandonment of the dichotomy between observed differences that are “real” and those that are “just due to chance” (18) – would be a radical solution to the problem we are concerned with here – the difficulties inherent in interpreting null findings from trials of ABI.

## Common Misunderstandings of Null Findings: The SIPS Project

As we have seen, despite its apparent shortcomings, NHST continues to be the preferred framework for investigation in much of psychology, psychiatry, and other branches of human science, and is certainly still prevalent in research evaluations of the effectiveness of ABI. (NHST as taught in textbooks today is a hybrid of the Fisher and the Neyman–Pearson approaches and no distinctions between these two approaches will be discussed here.) Opponents of NHST would no doubt attribute the misunderstandings of null findings that we will shortly consider to basic flaws in the logic of NHST (17, 18).

To illustrate these misunderstandings, we will focus on the so-called Screening and Intervention Program for Sensible drinking (SIPS) project in England. Other research on ABI could have been chosen for this purpose but SIPS is a recent and prominent evaluation, with potentially important implications for policy and practice and from which all the necessary points may be made. The project was funded by the UK Department of Health in 2006 following the publication of the Government’s Alcohol Harm Reduction Strategy for England (AHRSE) (19). In a section on Screening and Brief Interventions, the strategy said: “… the research evidence on brief interventions draws heavily on small-scale studies carried out outside the UK. More information is needed on the most effective methods of targeted screening and brief interventions, and whether the successes shown in research studies can be replicated within the health system in England…. The Department of Health will set up a number of pilot schemes by Q1/2005 to test how best to use a variety of models of targeted screening and brief intervention in primary and secondary healthcare settings, focusing particularly on value for money and mainstreaming” [(19), p. 43]. This led eventually to the funding of SIPS which consisted of a pragmatic, cluster-randomized controlled trial in each of three settings: PHC, accident and emergency services, and the criminal justice system. At the time of writing, only the results for the PHC trial have been published (20) and the other two trials will not be covered here. As was clear in the Government’s remit for this research stated above, the trials looked at issues to do with optimal forms of screening as well as effects of different modes of ABI but only the latter is of interest here.

The trial had a “step-up” design involving three groups in which components were successively added: (i) a control group consisting of the provision of a Patient Information Leaflet (PIL) together with the brief feedback of assessment results (i.e., whether or not the patient was drinking at a hazardous/harmful level); (ii) a brief advice (BA) group consisting of 5 min of structured advice about drinking plus the PIL; (iii) a brief counseling group (BLC) consisting of 20 min of counseling preceded by BA and followed by the PIL, and given to those patients who returned for a subsequent consultation. Across three areas of England, GPs and nurses from 24 practices that had not already implemented ABI were recruited and general practices were randomly allocated to one of the three conditions described above. Practices were incentivized to participate by payments amounting to £3,000 on successful completion of stages in the project. All primary care staff taking part in the trial were trained to deliver alcohol screening and brief intervention according to the trial protocol. Patients aged 18 or over routinely presenting in primary care and who screened positive on one of the screening instruments used in the trial were eligible for entry and a total of 756 were included. Analysis of outcomes at 6 and 12 months following intervention was by *intention to treat* which included all patients randomized to study groups whether or not they had been successfully followed up. Follow-up rates were 83% at 6 months and 79% at 12 months. Further details of the trial will be found in the protocol paper (21) and the main outcome paper (20).

**Figure 2:**
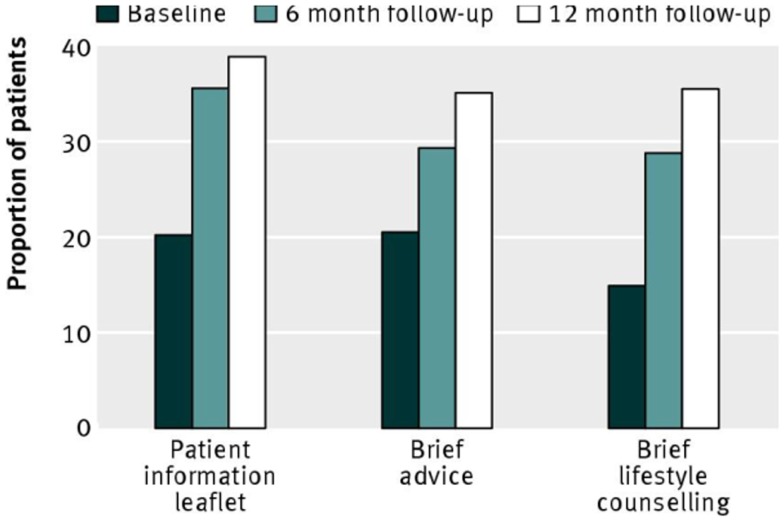
**Proportion of patients scoring <8 (negative status) on the alcohol use disorders identification test, representing non-hazardous or non-harmful drinking**. Reproduced from Kaner et al. [(19), p. 14].

With respect to interventions, the main hypothesis was that more intensive intervention would result in greater reduction in hazardous or harmful drinking, thus BLC > BA > PIL. In this context, and recalling the step-up design, the BA condition served as a control for the specific effects of BLC, the PIL condition served as a control for the specific effects of BA, and the PIL condition served as a control for the combined effects of BA and BLC). In the event, there were no significant differences between groups on the main outcome measure of the proportion of patients in each group who obtained a negative score on the Alcohol Use Disorders Identification Test [AUDIT, Ref. (22)]. This is shown by Figure 2, which gives these proportions at baseline, 6- and 12-month follow-up. Neither were there significant differences between groups on any other alcohol outcome measure [i.e., mean AUDIT score or extent of alcohol problems (23)]. A *per-protocol* analysis, which included only those patients who received a complete intervention and were successfully followed up, also failed to show any significant differences between groups.

The SIPS PHC trial was thus a well-designed and efficiently conducted investigation of the effects of two forms of brief intervention in real-world settings with adequate statistical power to detect an effect of brief intervention if one existed. The null findings were no doubt disappointing to the SIPS investigators and to many in the ABI field. But how should these null findings be interpreted or, of equal or possibly greater importance, how should they *not* be interpreted? We will now consider a number of ways in which the findings of the SIPS PHC trial have been misunderstood.

(i) The findings show that the three “interventions” under study are of equal effectiveness in reducing hazardous or harmful drinking.

This interpretation makes the classic error of “proving the null hypothesis” (24). The logic of NHST is based on the assumption that the null hypothesis is true. (The null hypothesis can be any specified difference between population parameters against which the research hypothesis is tested but in practice is almost always taken to be the “nil hypothesis” that the samples come from populations with identical parameters, e.g., that there is no difference between their means.) In a comparison of an experimental versus a control procedure, the NHST method gives the conditional probability of the occurrence of an experimental effect equal to or greater than that observed *given that the null hypothesis is true*. If that probability is sufficiently small at a preselected level, conventionally 0.05 or smaller, the null hypothesis is rejected and the alternative hypothesis that the samples come from different populations is accepted. However, NHST gives us no information whatever about the conditional probability of the null hypothesis being true *given the observed data* and to imagine that it does is one of the most common errors in the interpretation of the results of statistical tests [(17), Chapter 3]. If the probability of the observed difference is greater than the pre-set level for significance, all one can conclude is that one has failed to reject the null hypothesis, *not* that the null hypothesis has been proved or in any way supported. Put simply, it is not possible to prove something that has already been assumed. Note, however, that it is also fallacious to believe that the null hypothesis can eventually be “proved” by increasing the sample size and statistical power (25). Thus, with regard to the SIPS null findings, all that they should be interpreted as showing is that there is no evidence from this trial that the brief interventions under study are superior in effectiveness to their respective controls – “absence of evidence,” not “evidence of absence.”

In more practical terms, in addition to sampling variability and lack of statistical power, there may be many reasons for the failure to observe a statistically significant difference between experimental and control group means. It could be, for example, that the interventions, although shown to be efficacious in randomized controlled trials conducted in ideal research conditions, are not effective in more real-world conditions of routine practice (26) because they have not been faithfully implemented by the practitioners taking part in the trial (27) or because of some other difference between real-world conditions and the ideal research conditions in which efficacy was demonstrated.

One particular version of the “proving the null hypothesis” error focuses on the control condition in the SIPS trial and concludes that, since the PIL and assessment feedback making up that condition has been shown to be no less effective that the two successively more intensive brief interventions, this shows that the provision of an information leaflet combined with feedback of assessment results can substitute in practice for ABI. Indeed, this approach has been called “BI lite” (28). This issue will be returned to below.

Given that the fallacy of “proving the null hypothesis” is taught at an elementary level in courses on research methodology and statistics all over the world, it may be found surprising that such an error is frequently made in relation to the SIPS PHC findings. However, the present author can attest that this error is commonly encountered in commentaries on the SIPS findings in publications of various kinds, in papers given and conversations overheard at scientific conferences and other meetings, and in grant proposals seeking funding to pursue in some way the implications of the misinterpreted SIPS findings. Just one example comes from *Pulse*, a magazine for health professionals and which claims to be “at the heart of general practice since 1960” (29). This article is headed, “Patient leaflet enough to tackle problem drinking, researchers suggest” and begins “GPs should give patients with problem drinking a leaflet rather than advise them to reduce their alcohol intake.” This is because: “the SIPS study found informing patients of their drinking levels and offering a leaflet – handed to patients by a practice nurse – was just as effective as giving patient 5- or 10-min of lifestyle counseling.”

A possible contribution to this level of misunderstanding may be the fact that in some publications, the SIPS investigators described the trials as a comparison of the effects of “three intervention conditions” [e.g., Ref. (21)]. This may have led readers to view the before–after changes in consumption shown by control group patients as of interest in their own right and as a finding forming part of the evidence base relevant to the effects of ABI. What these changes mean will be discussed in the next section of this article but what can be said now is that the changes in the control group cannot be considered to be a “finding” about the effects of what was included in the control condition. At the risk of stating the obvious, any conclusion about these effects would have to be based on a comparison with a further non-intervention, assessment-only control group that did not include the PIL and/or assessment feedback, whichever of the two ingredients or their combination was thought to be of more interest. This was clearly recognized in the SIPS PHC outcome paper [(20), p. 5]. In view of the extensive evidence supporting ABI in general, the control condition used in the SIPS trials was the only kind likely to be found ethically acceptable. However, although the composition of the control group was perfectly defensible, to call it an intervention may have misled some consumers of the trial results and it would have been better to describe the trial in conventional terms as having two interventions that were evaluated in comparison to a control condition.

(ii) The PIL plus assessment feedback has been shown to be more cost–effective than BA and brief counseling and should therefore be implemented in practice.

This common misinterpretation is clearly related to the previous one but has more direct and very misleading implications for practice. It is certainly true that the provision of a leaflet together with information about assessment results would be cheaper to implement than either of the two forms of ABI because it would take less time and would require much less training to deliver. However, the conclusion that it would be less costly, even statistically significantly so, is all that can be claimed and, indeed, all that was claimed by the SIPS investigators (20). The underlying mistake is to infer that, because the three “interventions” were equally effective, then the less costly one must be more cost–effective but, as we have seen, it cannot be concluded that the ABI and control conditions were equally effective. And something cannot be called cost–effective if there is no evidence that it is effective in the first place.

(iii) The reductions in consumption shown in all three groups were caused by the “interventions” participants had received.

Again, this misunderstanding is closely related to the two previously described. The phenomenon in question will be explored in detail in the following section. Here though it can be noted that, by the logic of experimental research, in order to make a causal inference of this kind it is necessary to show that reductions in drinking shown in the ABI groups were statistically significant larger than those shown in their appropriate controls and this was obviously not the case. With regard to the control group reductions, as noted above, there was no appropriate further control for the effects of the ingredients of the SIPS control group, so no causal inferences of any kind may be made. Thus, there was no evidence from the SIPS PHC trial that any of the conditions under study led to changes in participants’ drinking.

It should be stressed that the importance of these misunderstandings is not limited to academic debates between scientists in learned journals; they could well affect the future provision of ABI in England and perhaps in other countries. It is well known that there have been considerable difficulties in persuading GPs, nurses, and other healthcare professionals to implement ABI routinely in their practices; there is a copious literature on this problem (30) and how it may be redressed (31). In surveys of health professionals’ attitudes to this work, one of the most commonly encountered obstacles is “lack of time” or “too busy” (32, 33). There has also been resistance in England to the inclusion of ABI in the NHS *Quality and Outcomes Framework*, under which general practices are reimbursed for preventive activity. This has created considerable pressure on the relevant sections of the Department of Health in London (and now its replacement body for this area of work, *Public Health England*) to make the interventions that health professionals are being encouraged to implement as short and easy to deliver as possible. So too, given the multitude of demands on their time from a large number of health bodies, it would be expected that many GPs would call for ABI to be whittled down to more manageable forms. In times of austerity, the appeal of shorter, simpler, and less expensive interventions for widespread implementation in practice must be seductive to policy-makers.

It is little wonder then that the misunderstandings of the SIPS findings listed above have been used to recommend the provision in practice of a PIL as a substitute for ABI, as in the *Pulse* article mentioned above. At the risk of repetition, it is not being argued here that this minimal kind of intervention would necessarily be ineffective, merely that there is no good evidence at present that it *would* be effective. If it is ineffective, or substantially less effective than ABI proper, and even if GPs and practice nurses definitely prefer it, its roll-out would represent a waste of precious resources. And before its ineffectiveness is clearly demonstrated, it might also derail the effort to achieve the full implementation of ABI proper that is necessary for widespread clinical benefit and put back the prospect for achieving this implementation by many years.

It might be conceded that the offer of a PIL following an assessment of alcohol-related risk and harm and the feedback of the results of that assessment could be defended on purely pragmatic *a priori* grounds. Given that resources to implement ABI proper are scarce and that most GPs and nurses are unwilling to implement anything more intensive, given too the principle that it is unlikely to do harm and may even do some good – perhaps starting a process of contemplating the need for change that might eventually lead to action to cut down drinking (34) – this could amount to a justification for implementing this minimal intervention (28). The claim would be that it must surely be better than nothing. But however it is justified, it should not be by a fallacious inference from the findings of the PHC arm of the SIPS trial.

## Why Do Control Groups in Trials of Alcohol Brief Intervention Show Reductions in Mean Consumption?

Control groups in trials of ABI frequently show reductions in mean alcohol consumption from baseline to follow-up and this was certainly the case in the SIPS PHC trial (see Figure 2). In a review of such trials, it was calculated that control group participants reduce their drinking by approximately 20% (35, 36). A reduction in drinking of this size is larger than overall differences between experimental and control groups at follow-up (2) and it is a reasonable assumption that reductions in control groups of this order may prevent the true effects of ABI from being observed (37). We also saw that the reductions in consumption shown by control group participants in the SIPS trial (or, rather, the increase in the proportion of participants not showing hazardous/harmful drinking – see Figure 2) has been wrongly assumed to have been *caused* by the control group procedures, i.e., the provision of a PIL and/or the feedback of assessment results. To clarify further why it is a mistake to make this inference, we will now consider other possible reasons for reductions in control group consumption. In recent times, our understanding of these reasons had been greatly assisted by the work of Dr. Jim McCambridge of the London School of Hygiene and Tropical Medicine and his various colleagues.

### Regression to the mean

This must be one of the most misunderstood concepts in health care science (38). It is often thought that because, for example, participants in a trial of an alcohol intervention are recruited at a particularly high point in their alcohol consumption, they make a decision to try to cut down drinking, which is reflected in their lower consumption at follow-up. This is incorrect; regression to the mean is a purely statistical phenomenon with no reference whatever to decisions by trial participants or any other causal factor impinging on the outcome variable of interest.

Regression to the mean can be thought of as the obverse of correlation (39). If any two randomly distributed properties of individuals are less than perfectly correlated in a population, then it must be the case that extreme scorers on one of the variables will tend to show less extreme scores on the other. This applies in both directions; high scorers on the first variable will tend to show lower scores on the second and low scorers on the first will tend to show higher scores on the second. The smaller the correlation between the two variables, the greater will be the tendency for those with more extreme scores on one variable to approach the mean in their scores on the other. In the example in which we are interested, the two variables in question are the same participants’ scores on the AUDIT questionnaire (22) at entry to the trial and at follow-up. In this case, however, participants will have been selected for entry to the trial on the basis of their relatively high scores (i.e., above the recognized cut-point for hazardous/harmful drinking) on the AUDIT. As a consequence, it is inevitably true that participants’ scores at follow-up will tend to be lower than at intake due only to the nature of random fluctuation and statistical correlation. The same applies to any variable used for trial selection that is correlated, but less than perfectly so, with a variable used to evaluate outcome at follow-up.

The possible effects of regression to the mean on control group participants in brief intervention trials were studied empirically by McCambridge and colleagues (40). These authors gave the AUDIT to a large cohort of university students in New Zealand at baseline and 6 months later, without any attempt to intervene in their drinking. Selecting from this cohort for analysis those individuals with a baseline AUDIT score of 8+, the usual cut-point for entry to trials of ABI, the observed mean reduction over time was approximately half that obtained in the full sample without selection. When selection was made using a series of higher AUDIT thresholds, the observed reductions in mean alcohol consumption were successively larger. This evidence suggests that a substantial part of the reduction in consumption shown by control groups can be explained by the statistical artifact of regression to the mean.

### Research participation effects

This is an umbrella term referring to a range of ways in which merely taking part in a research study can influence participants’ behavior, quite apart from any effects on behavior the researchers may intend (41). An older term for these influences is “Hawthorne effects,” referring to a famous series of studies from 1924 to 1933 at the Hawthorne Works of *Western Electric* outside Chicago. The results of these studies were interpreted as showing that the productivity of workers increased just through their awareness of having their behavior monitored as part of a research project, although other explanations are possible (42). In a systematic review of the literature relevant to the Hawthorne effect (43), it was concluded that the effect certainly existed but that little could be confidently known about it, including how large it was, without more research.

The wider term “research participation effects” refers to a range of phenomena that might introduce bias in estimates of behavior change in randomized controlled trials. These include the effects of signing an informed consent form and of reactions to randomization – for example, disappointment or resentment at being allocated to the control rather than the intervention condition. The possible effects on behavior of being screened or assessed prior to randomization will be considered below. Another important class of research participation effects is known by psychologists as “demand characteristics” (44). This refers to expectations participants may have about what the researcher is interested in studying and possible attempts by them to conform, or not, to what they think the researcher is trying to demonstrate. This source of bias is mainly relevant to laboratory research but McCambridge and colleagues have reviewed evidence of its possible influence on participant behavior in non-laboratory settings (45). An obvious example here is a tendency by a participant at research follow-up to underestimate their alcohol consumption because they surmise that the project is trying to reduce this outcome and they wish to please the follow-up interviewer; alternatively, they might exaggerate their consumption in a deliberate attempt to undermine what they guess is the purpose of the project. Influences of this sort could apply both to control and intervention group participants and represent one kind of problem with the validity of self-reports of behavior in research trials.

### Historical trends

An obvious way in which the alcohol-related behavior of control group participants might be influenced is by changes over time in the *per capita* consumption of alcohol in the geographical area in which the research is taking place. Average consumption at follow-up compared with trial entry could be reduced due to the increased price of alcoholic beverages, through higher taxation or in other ways, which is known to be strongly related to consumption levels (46). Changes in the density of alcohol retail outlets, community attitudes to drunkenness, stricter enforcement of drink-driving legislation and a large number of other variables that can affect the level of alcohol consumption in a population (47) could also contribute to these reductions.

### Assessment reactivity

This last category of possible explanations for control group reductions in consumption has been the one to which most attention has been devoted in the literature on ABI. The idea here is that simply requiring a research participant formally to answer questions about their drinking can affect the drinking itself (48). This might be by directing participants’ attention to their drinking and raising the possibility in their minds that it might be hazardous or harmful, thus leading to attempts to cut down, or in some other unknown way. The literature has focused on the effects of research assessment conducted after informed consent has been obtained, which can sometime take longer to complete than the ABI itself (49), but the effects of screening carried out prior to informed consent and entry to the trial have also been examined (50). Possible screening effects will be included under “assessment reactivity” in the remainder of this discussion.

McCambridge and Kypri (51) conducted a systematic review and meta-analysis of studies in the field of ABI that had attempted to answer the question of whether and by how much research assessments influence behavior by using randomized experimental methods. Ten studies were identified, of which eight provided findings for quantitative analysis. The general conclusion of this review was that research assessment did alter subsequent self-reported behavior in relation to alcohol consumption but that the effect was small, equivalent to 13.7 g of ethanol per week (one US standard drink or 11/2 UK units). On the other hand, as the authors point out, although small, this effect amounted to about 35% of the most recent and reliable estimate of the effect of ABI itself (2).

Of the eight studies included in the meta-analysis (51), five took place in university student populations and might be considered less than fully relevant to the matter at hand here. The three studies that took place in health care settings included two in emergency departments (52, 53) and one in PHC (54). None of these studies reported significant effects of assessment (or, indeed, of ABI). It is obvious that we need more studies of this kind to arrive at reliable estimates of the effects of assessment on subsequent drinking but at present it appears that such effects are smaller in health care than in university student settings.

McCambridge and colleagues subsequently conducted a study in Sweden (the AMADEUS Project) (55) to evaluate the effects of online assessment and feedback of results from the AUDIT-C (56). University students were randomized to groups consisting of (i) assessment and feedback; (ii) assessment-only without feedback; and (iii) neither assessment nor feedback. Findings were that students in group (i) had significantly fewer risky drinkers at 3-month follow-up than those in group (iii), while students in group (ii) scored lower on the AUDIT-C at follow-up that those in group (iii). This study thus provided some evidence for the effects of assessment and feedback on drinking behavior but findings were short-term and inconsistent, and the effects themselves small.

To return to a consideration of the SIPS primary care findings, it is sometimes suggested that a mere assessment of someone’s drinking can serve as well as an ABI or, at least, will result in a reduction in alcohol consumption that would be valuable in busy health care settings with little time to do much else. The notion that research assessments could be the ABIs of the future has received serious attention (57). There are several points to make here. First, we have just seen that the evidence to support this suggestion is very thin; more research may reveal a different picture but, at present, there is insufficient evidence to conclude that assessments, at least of the kind normally used in research, can substitute for ABI as it has traditionally been conceived in health care settings. Secondly, although they may have the effect of inducing behavioral change by drawing attention to drinking, questions making up conventional research assessment are not designed explicitly to promote such change, e.g., by deliberately seeking to foster a discrepancy between the person’s actual self-concept in relation to drinking behavior and the drinking of their ideal self, by asking explicitly about intentions to cut down or quit, or by enquiring about the perceived benefits of more moderate drinking (51, 58). Thus, future research might evaluate the effects of assessments of alcohol-related behavior deliberately designed to encourage changes in drinking. Thirdly, an appropriate research design for the investigation of the effects of assessment reactivity would be a non-inferiority trial (59) in which an assessment-only condition is compared to an ABI with the hypothesis that it is not inferior in its effects on drinking at follow-up. Using the methodology and recommended analysis for a non-inferiority trial, it would be possible to show that two types of intervention do not differ in effectiveness.

Lastly, the suggestion that assessments might serve to reduce drinking says nothing about the possible effects of feeding back assessment results or of providing a PIL. If it is true that assessments are effective in themselves, the contents of the control condition in the SIPS trial might be entirely redundant and need not be part of an effective intervention. On the other hand, it is reasonable to think that assessment feedback *would* make an additional contribution to change and that giving the patient information to take away that could be consulted if the motivation to change increases might also be an effective ingredient of intervention. In the first case, assessment feedback forms an essential part of a type of intervention known in different circumstances to be effective (60), albeit over two sessions, and is also integral to *Motivational Enhancement Therapy* (61), albeit over three or four sessions. In relation to the provision of a PIL, and depending on how much information of what kind it contained, bibliotherapy in general has been shown to be an effective means of decreasing alcohol problems (62). The truth, however, is that we do not know if assessments, assessment feedback or PILs are effective in themselves or in combination, and it is to these questions that research should be directed.

It will not have escaped the reader’s attention that all four possible explanations above for reductions in alcohol consumption in control groups in trials of ABI apply equally well to reductions in intervention groups in those trials. It is precisely for that reason that, if we wish to make real progress in implementing effective ABIs in routine practice, we cannot avoid relying on randomized trials in which these factors are controlled across intervention and control groups, leaving the only difference between groups the intervention component under test. However, plausible current inferences from the literature may seem in which a case is made for the widespread introduction of assessment feedback and PIL as a substitute for ABI proper, there is no way such a policy can pretend to be evidence-based. If they believe at all in evidence-based practice, those who favor the implementation of screening followed by simple feedback and written information must be able to show that such a procedure is superior in effectiveness to appropriate control conditions in well-designed and sufficiently powered pragmatic randomized controlled trials. To implement this procedure without such evidence risks wasting hard-fought gains of 30 years research on ABI.

## Distinguishing between Absence of Evidence and Evidence of Absence

We saw above that, under the conventional NHST approach to statistical inference from RCTs, when no significant differences on outcome measures between intervention and control groups have been found, we are unable to distinguish between two conceivable interpretations of these null findings: (i) there is no evidence that the means of the two groups differ and nothing can be said about the effectiveness of intervention one way or the other, and (ii) there is evidence that the means do not differ, that the null hypothesis is true and that the intervention is therefore ineffective. These two interpretations have been shortened here to (i) absence of evidence and (ii) evidence of absence. This dilemma can be applied, of course, to more than one experimental group in comparison to a control group, as in the SIPS PHC findings discussed above. It is this dilemma, so this article has argued, that has held back, and continues to hold back, progress in a scientific understanding and beneficial application of ABI.

There are two sets of unfortunate possible consequences of this lack of information. First, in the situation where absence of evidence is properly concluded from non-significant findings but there is actually no difference between means in the population, time and resources may be wasted on continuing to search for an effect of intervention when none in fact exists. On the other hand, if it is improperly concluded under the NHST approach that there is evidence of an absence of difference between means when there is in fact a real potential effect of intervention in the population, then an opportunity to implement, or at least to support the implementation of, an effective intervention will have been missed. Both these kinds of negative consequence may have interfered with progress on particular forms of ABI in the past. More important from the present perspective, they are likely to retard research on the effects of ABI in the many novel populations of hazardous and harmful drinkers in which it is desired to implement ABI and the novel settings in which these drinkers may be found.

There is, however, a solution to this problem but it means abandoning the NHST handling of null findings in favor of an approach from Bayesian statistics. The Bayesian approach to the problem of interpreting null findings has been developed recently by Dr. Zoltán Dienes of the University of Susses (63) and this section will rely heavily on his work. This is not the place to attempt a complete description of Bayesian statistics but good introductions are available (64, 65), including one by Dienes (66) comparing the Bayesian approach to statistical inference by the orthodox approach.

Suffice it to say here that Bayesian statistics is founded on a completely different philosophical understanding of probability from conventional NHST statistics. Bayesian statistics defines probability *subjectively*, as a measure of the degree of confidence one has that some event will occur or that some particular hypothesis is true. The conventional, Neyman–Pearson approach on which NHST is based defines probability *objectively*, in terms of long-run relative frequencies of the occurrence of events. From this fundamental difference in the understanding of probability all other differences flow. The mantra of Bayesian statistics is: “the posterior is proportional to the likelihood times the prior.” Working backwards, the “prior” is the subjective probability that a hypothesis is true before collecting data; the “likelihood” is the probability of obtaining the observed data given that the prior hypothesis is true; the “posterior” is the probability of the hypothesis being true given the observed data and is calculated by multiplying the likelihood by the prior. From the Bayesian perspective, scientific progress consists of updating the probability of hypotheses being true in the light of observed data (66).

While under NHST only two conclusions are possible from the results of an experiment, either the null hypothesis is rejected or it is not, from a Bayesian perspective there are three: (i) there is strong evidence for the alternative hypothesis; (ii) there is strong evidence for the null hypothesis; (iii) the data are insensitive with respect to the alternative and null hypotheses. To determine which of these conclusions applies to any given sets of results, it is necessary to calculate something called the *Bayes Factor* (B). This is the ratio of the likelihood of the observed data given the alternative hypothesis over the likelihood of the data given the null hypothesis. If this ratio is >1, the alternative hypothesis is supported; if it is <1, the null hypothesis is supported; and if it is about 1 the experiment is insensitive and neither hypothesis is supported. To arrive a firm decision in practice, recommended cut-offs (67) are that B >3 represents substantial evidence for the alternative hypothesis and B less that 1/3 represents substantial evidence for the null hypothesis, with values in between representing a range of weak evidence for either hypothesis depending on whether B is greater or less than 1.

One immediate advantage of the Bayesian method is that the researcher is forced to stipulate an alternative hypothesis in terms of the size of the effect that, say, an intervention is expected to show relative to a control condition and its minimum and maximum values. While the stipulation of the alternative hypothesis is often said to be desirable under NHST, it is rarely done. In practice, the Bayesian researcher specifies a range of population values for the parameter of interest, say the difference between intervention and control group means, with prior probabilities for each population value and the way in which these probabilities are distributed over the range of population values [(66), Chapter 4]. This procedure facilitates good science.

It will have been noted that, although the Bayesian approach allows the null hypothesis to be accepted, there is still an intermediate range of values of B, conventionally between 1/3 and 3, where the evidence is weak and which can therefore be considered a reappearance of the absence of evidence conclusion. However, the striking difference between Bayes and NHST in this situation is that, in the former, the researcher can quite legitimately continue to collect data until one of the two boundary conditions, either 3 or 1/3, is reached; this is the only “stopping rule” that applies to data collection under Bayes. By contrast, under NHST the collection of further data beyond the sample size given by the power calculation and stipulated before the experiment began is methodologically spurious and, if not openly declared, unethical. Of course, owing to the finite nature of research funding, fixed research plans and other practical matters, it will often be impossible to collect more data but the opportunity remains available in principle under the Bayesian method. And it is important to repeat that, even if further data collection is not possible, the information deriving from the Bayesian approach is still superior to that from NHST in allowing the distinction to be made between evidence of absence and absence of evidence.

In more general terms, the battle for dominance between Bayesian and Fisher/Neyman–Pearson statistical inference has been waged for many years between camps of statisticians, philosophers, and those researchers who take an interest in the fundamentals of their scientific disciplines (68). Those who favor Bayes, and have described its varied advantages over conventional statistics, have found that change in scientific practice, especially in the human sciences, is slow to occur. Journal editors, for example, may be loath to accept papers based on Bayesian statistics and, in any event, Bayesian and conventional analyses will often agree in their conclusions. As Dienes (63) points out, however, one way in which they do clearly disagree is in the interpretation of non-significant results. The solution here is to use mainly orthodox statistics but, whenever a non-significant result is found, to calculate a Bayes factor in the interest of disambiguation. This seems an eminently sensible solution to the problem of null findings which, as has been argued in the article, holds back progress in the field of ABI research. A program for calculating Bayes Factors can be accessed at http://www.lifesci.sussex.ac.uk/home/Zoltan_Dienes/inference/Bayes.htm.

If this solution were adopted, when we observed a non-significant result from an RCT, it would be possible to conclude that the specific form of ABI being evaluated was ineffective and not worth pursuing further, so that precious resources would not be wasted. On the other hand, we could conclude that it was unclear whether the ABI in question was effective or not and that further research was needed. The difference from the conclusion based on the conventional perspective, however, is that we would already have ruled out the possibility that the intervention was ineffective. [It is also possible that the Bayes Factor could provide evidence for the alternative hypothesis and allow the conclusion that the intervention was effective when the conventional NHST approach had not been able to reject the null hypothesis (63).] This method could be applied to the non-significant results of trials such as SIPS to reduce uncertainly about and possible misunderstanding of their results. The results of an analysis of SIPS data using the Bayesian approach to null findings will form the basis of a further communication.

## Conflict of Interest Statement

The present author was a Principal Investigator on the SIPS trial that is discussed in this article and an author on papers arising from it. He has no other possible conflicts of interest to declare.

## Footnotes

^1^http://www.youtube.com/watch?v=ez4DgdurRPg&feature=youtu.be

^2^http://www.latrobe.edu.au/psy/research/cognitive-and-developmental-psychology/esci
